# Bacterial response to spatial gradients of algal-derived nutrients in a porous microplate

**DOI:** 10.1038/s41396-021-01147-x

**Published:** 2021-11-17

**Authors:** Hyungseok Kim, Jeffrey A. Kimbrel, Christopher A. Vaiana, Jessica R. Wollard, Xavier Mayali, Cullen R. Buie

**Affiliations:** 1 Department of Mechanical Engineering, Massachusetts Institute of Technology, Cambridge, MA USA; 2 Institute for Data, Systems, and Society, Massachusetts Institute of Technology, Cambridge, MA USA; 3 Physical and Life Science Directorate, Lawrence Livermore National Laboratory, Livermore, CA USA; 4 Department of Biological Engineering, Massachusetts Institute of Technology, Cambridge, MA USA

**Keywords:** Microbial ecology, Microbial ecology, Microbial ecology

## Abstract

Photosynthetic microalgae are responsible for 50% of the global atmospheric CO_2_ fixation into organic matter and hold potential as a renewable bioenergy source. Their metabolic interactions with the surrounding microbial community (the algal microbiome) play critical roles in carbon cycling, but due to methodological limitations, it has been challenging to examine how community development is influenced by spatial proximity to their algal host. Here we introduce a copolymer-based porous microplate to co-culture algae and bacteria, where metabolites are constantly exchanged between the microorganisms while maintaining physical separation. In the microplate, we found that the diatom *Phaeodactylum tricornutum* accumulated to cell abundances ~20 fold higher than under normal batch conditions due to constant replenishment of nutrients through the porous structure. We also demonstrate that algal-associated bacteria, both single isolates and complex communities, responded to inorganic nutrients away from their host as well as organic nutrients originating from the algae in a spatially predictable manner. These experimental findings coupled with a mathematical model suggest that host proximity and algal culture growth phase impact bacterial community development in a taxon-specific manner through organic and inorganic nutrient availability. Our novel system presents a useful tool to investigate universal metabolic interactions between microbes in aquatic ecosystems.

## Introduction

Metabolic interactions between microalgae and their associated bacteria, the latter sometimes referred to as the algal microbiome, have been recognized as an important contribution to carbon cycling in natural [1] and engineered [2] algal-dominated ecosystems. Heterotrophic bacteria consume up to 50% of the carbon fixed by algae [3], and this mineralization process often leads to an exchange of metabolites from bacteria to algae, providing a variety of micronutrients such as trace metals [4], vitamins [5–7], and phytohormones [8], which can be scarce in nature yet are essential for algal growth. Due to the potential influence of the algal microbiome on algal physiology, much effort has been made to identify algal-associated bacterial community structure and understand how it is related to algal diversity. In particular, it has been revealed that the algal-associated bacterial community can be highly conserved across time [9, 10], uniquely shaped by their algal host [11–13], and exhibits structural differences between algal-attached and free-living [13].

These studies indicate that the community-level bacterial response to algal hosts depends at least partially on physical proximity, including physical attachment, as well as the type of algal-excreted products. Maintaining physical proximity can significantly enhance the exchange rate of metabolites between microorganisms, which is especially important under an open-ocean environment where the nutrients are scarce [14] and marine aggregates can contribute to the majority of nutrient cycling [15]. Indeed, microscale interactions between algae and bacteria are commonly observed as direct cell-to-cell attachment [11, 12, 16, 17] or bacterial chemotaxis towards local gradients of algal exudates [18–21], with ongoing efforts to visualize these processes via micro or nano-scale chemical imaging [22, 23].

The predominant mechanism that explains how heterotrophic bacteria respond to algal-derived organic matter is the diffusive transport of metabolites. Metabolites diffusing around an alga create a spatial gradient of nutrients, and the region where the algal-derived organic nutrients are abundant is termed the phycosphere [1, 21, 24]. For an alga sized less than 100 µm, diffusion within its phycosphere plays a major role in transporting metabolites rather than fluid advection [1] or turbulence [25]. Moreover, diffusive transport is considered to be 50 times more efficient for encountering molecules than cellular swimming, especially at a low stirring number (defined as *lv* / *D*; *l* is intermolecular spacing, *v* swimming speed, and *D* diffusion coefficient) [14, 26]. Therefore, by maintaining spatial proximity to the algal cell inside the phycosphere, bacteria can best exploit algal-derived organic matter through diffusive effects.

In order to assess how microorganisms respond to the diffusion of metabolites such as algal exudates, in vivo co-culture methods have been developed. For example, a dual-chamber system separated by a porous membrane [20, 27–29] has enabled further downstream biological analyses, such as plate reader-compatible in situ growth measurements [28], metabolomics [27], or microscopy to track cellular motility [20] and biofilm formation [29]. Microscopy has indeed provided valuable information on how bacteria can temporally respond to the presence of algae via chemotaxis [19, 20]. Nonetheless, existing techniques have not yet enabled the co-cultivation of microalgae with multiple bacterial species, especially for time periods long enough to detect community-level dynamics (days to weeks).

**Fig. 1:**
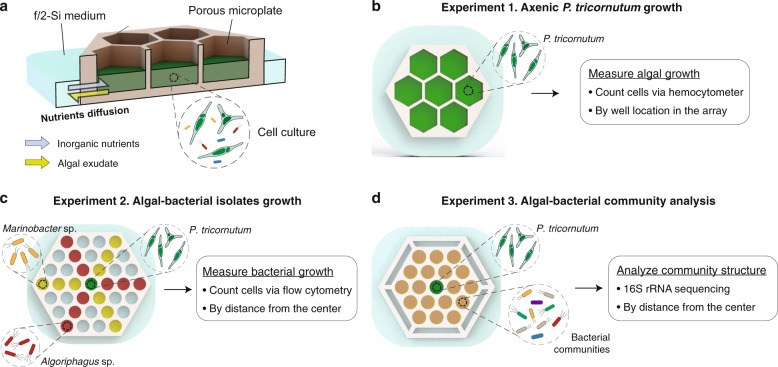
Porous microplate for algal-bacterial interaction. **a** Cross-sectional schematic illustrating cell culture in a porous microplate. **b**–**d** Three experimental designs showing a top-view schematic of microplate wells to measure the growth of (**b**) *P. tricornutum*, (**c**) bacterial isolates, and (**d**) to analyze community structure of algal-associated bacteria. To avoid cross-contamination between adjacent wells in the co-culture microplate, each well was designed with a rounded shape as the polygonal shape generates a sharp liquid meniscus near the top of the microplate [63].

In this paper, we present a porous microplate targeted to the study of in vivo interactions between algae and free-living bacteria. We develop a protocol to culture the microorganisms for up to several weeks in the porous microplate, overcoming previous limitations mentioned above. We employ the copolymer poly(2-hydroxethyl methacrylate-*co*-ethylene glycol dimethacrylate) (HEMA − EDMA), previously shown to be an effective building copolymer to enable cell-to-cell communication ranging from microbial [30] to mammalian cells [31]. Using this approach, we set our central goal to understand how metabolite diffusion plays a role in the interaction between algal cells and their associated bacteria. We explore how free-living bacterial populations respond to algal exudate diffusion by altering its input to bacterial communities through changing the physical proximity between the source (the algae) and the sink (the bacteria). Specifically, by taking advantage of our incubation system (Fig. 1a), we hypothesize that inorganic nutrients from the medium are converted into dissolved organic carbon (DOC) through algal photosynthetic activity in vivo and that the conversion creates a spatial gradient of DOC and inorganic nutrients across the culture wells, the response to which can be detected by changes in bacterial abundance and community structure.

To test our hypotheses, we designed three consecutive experiments with algae and free-living bacteria incubated in the porous microplate system. We first examined the model alga *Phaeodactylum tricornutum* grown in porous microplate wells that are spatially arranged to generate different nutritional availability and quantify growth (Fig. 1b). Next, we co-cultured *P. tricornutum* with two commonly algal-associated bacterial strains (of the genera *Algoriphagus* and *Marinobacter*), where the bacteria were incubated at different distances from the alga (Fig. 1c). These bacteria were previously isolated from *P. tricornutum* mesocosms [11, 16] and have been shown to affect algal growth: *Marinobacter* increased the cell size, abundance and lipid content of *P. tricornutum*, and *Algoriphagus* incorporated more of the *P. tricornutum* exudates than *Marinobacter* [32]. Although the strains are phycosphere-associated, they rarely or do not attach to *P. tricornutum* (Supplementary Fig. S1), thereby allowing us to use them as free-living model organisms targeted to study certain aspects of the phycosphere interaction. Finally, we incubated *P. tricornutum* in the porous microplate with mixed bacterial communities to observe how community structure responds to physical distance away from the algal host (Fig. 1d).

## Materials and methods

### Acrylic and polydimethylsiloxane (PDMS) molds preparation

**Fig. 2:**
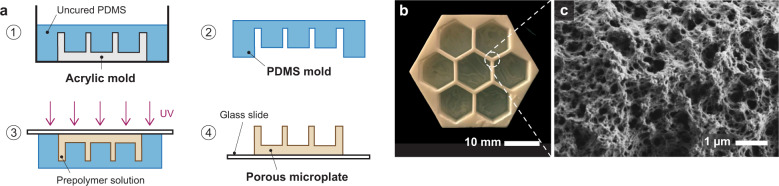
Synthesis and characterization of porous microplate. **a** Procedure to build a porous microplate using polydimethylsiloxane (PDMS) and acrylic molds. **b** Image of the microplate with an array of culture wells (wall thickness: 0.9 mm). **c** Scanning electron microscopy image of nanoporous copolymer HEMA–EDMA.

The incubating device for the porous microplate was designed using a CAD software (Solidworks, Dassault Systèmes) and the exported drawing files were used to laser cut 1/4'' and 1/8'' acrylic sheet (Universal Laser Systems; Supplementary Fig. S2). After washing the cut acrylic parts with deionized water, they were attached by acrylic (Weld-On) and epoxy (3 M) adhesives that were followed by a curing process for ~18 h. Polydimethylsiloxane (PDMS) (Sylgard 184, Dow Corning) was cast onto the acrylic mold and cured at 80 °C for at least 3 h. The PDMS mold was carefully detached from the acrylic surface by dispensing isopropyl alcohol (VWR) into the area between the PDMS and the acrylic molds (Fig. 2a).

### Porous microplate preparation

Synthesis of copolymer HEMA–EDMA was based on previously described protocols [30, 31] and details are given as follows. Prepolymer solution HEMA − EDMA was prepared by mixing 2-hydroxyethyl methacrylate (HEMA; monomer, 24 wt.%, Sigma-Aldrich), ethylene glycol dimethacrylate (EDMA; crosslinker, 16 wt.%, Sigma-Aldrich), 1-decanol (porogen, 12 wt.%, Sigma-Aldrich), cyclohexanol (porogen, 48 wt.%, Sigma-Aldrich) and 2,2-dimethoxy-2-phenylacetophenone (DMPAP; photoinitiator, 1 wt.%). The solution was stored at room temperature without light exposure until further use. Glass slides (75 × 50 mm^2^, VWR) were chemically cleaned by sequentially soaking in 1 M hydrochloric acid and 1 M sodium hydroxide for one hour, followed by rinsing with deionized water and air drying. The prepolymer solution was cast onto the PDMS mold and a glass slide was placed on the mold. The solution was then polymerized under ultraviolet light with a wavelength 365 nm for 15 min by using a commercial UV lamp (VWR). The photopolymerized device was detached from the PDMS mold and stored in a jar containing methanol (VWR) until further use (Fig. 2a). The jar was refilled with new methanol twice in order to remove the remaining porogen and uncrosslinked monomers from the hydrogel.

Upon each incubation experiment with the porous microplate, each device was decontaminated by replacing the solvent with 70% alcohol (VWR) and storing it for 24 h. They were immersed in a pre-autoclaved jar for two weeks with f/2 medium with omitted silicate, where the jar was refilled once with a new sterile medium to adjust its pH for the algal culture and remove any solvent remaining in the hydrogel. Before inoculating microbial cells, each microplate was taken out from the jar and the media remaining on the top surface was removed by absorbing it with a pre-sterilized wipe to minimize the chance for cross-contamination between wells (Fig. 2b).

### Scanning electron microscopy

Photopolymerized HEMA − EDMA was removed from methanol and dried in air for at least one week to evaporate the excess solvent. A ~5 × 5 mm^2^ specimen was collected from the dried copolymer and attached to a pin stub. The stub was loaded on a scanning electron microscope (SEM; MERLIN, Carl Zeiss), and the specimen was characterized with imaging software (SmartSEM, Carl Zeiss) with 16,270X magnification and an operating voltage of 1 kV. The SEM imaging was performed at the Electron Microscopy Facility in the MIT Materials Research Science and Engineering Centers (MRSEC; Fig. 2c).

### Strains and culturing conditions

Axenic *P. tricornutum* CCMP 2561 was acquired from ﻿the National Center for Marine Algae and Microbiota (NCMA) and shown to be axenic via epifluorescence microscopy and sequencing of the 16 S rRNA gene [11]. *P. tricornutum* was maintained in f/2 medium with 20 g L^−1^ commercially available sea salts (Instant Ocean, Blacksburg) and with omitted silicate, which we will refer to as f/2-Si [11, 16]. Batch cultures were grown at 20 °C with a 12 h light/12 h dark diurnal cycle and a light intensity of 200 μmol photons m^−2^ s^−1^ (Exlenvce). Every 2–3 weeks, axenic cultures were monitored for bacterial contamination by streaking culture samples on marine broth agar [33], that tests for contamination by bacteria that can grow on agar media and is not definitive. Every 6–12 months, every axenic and bacterial co-culture of *P*. *tricornutum* was inspected for the absence/presence of bacteria by staining the cellular DNA with 0.1% v/v SYTO BC Green Fluorescent Acid Stain (Thermofisher, Supplementary Fig. S1).

Bacterial community samples (referred to as “phycosphere enrichments”) were obtained from mesocosms of *P. tricornutum* and maintained as previously described [11, 16]. Briefly, an outdoor *P. tricornutum* mesocosm sample in natural seawater was collected in Corpus Christi, TX and filtered with 0.6–1 µm pores to remove larger algal cells. The bacterial filtrates were inoculated to an axenic algal culture, maintained in f/2-Si media for ~3 months, and washed with a sterile medium to enrich for phycosphere-associated bacteria. These enriched communities were subsequently co-cultured with *P. tricornutum* in f/2-Si media for ~4 years prior to the start of the experiments.

Two bacterial strains, *Marinobacter* sp. 3-2 and *Algoriphagus* sp. ARW1R1, were isolated from the phycosphere enrichment samples (Supplementary Table S1). The isolates were either maintained by growing on marine broth agar plates at 30 °C or by co-culturing with *P. tricornutum* through inoculation of a single colony into the axenic culture.

### *P. tricornutum* culture in porous microplate

Three baseline experiments were designed to study how the alga *P. tricornutum* interacts with its associated bacteria in the porous microplate (Fig. 1). For experiments assessing the algal growth in the microplate, axenic *P. tricornutum* was acclimated to a copolymer environment in advance by inoculating a stationary phase-culture to a separate microplate. After acclimation for 4 days, the culture was diluted to ~1 × 10^6^ cells ml^−1^ and transferred to the experimental microplate. Three replicated microplates were placed in a single transparent covered container (128 × 85 × 10 mm^3^, VWR) which was filled with ~25 ml f/2-Si medium to keep the microplate hydrated throughout the incubation period of 20 days with an initial culture volume of 75 µl (Fig. 1a). The procedures were conducted under a biosafety cabinet to prevent any biological contamination. The cells were incubated under the same conditions as described above for the batch cultures (temperature, light intensity, diurnal cycle).

**Fig. 3:**
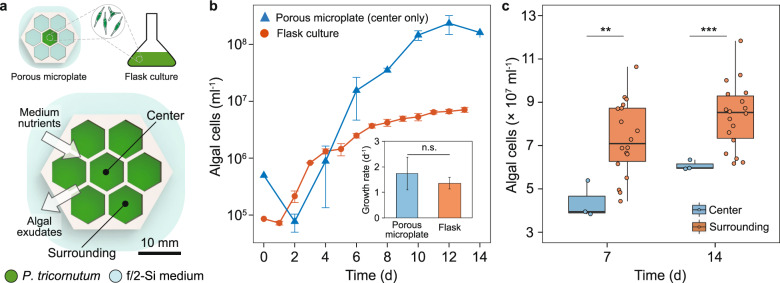
Cultivation of *P. tricornutum* in the porous microplate. **a** Schematic of a microplate for algal cultivation. **b** Growth curve and maximal growth rate (inset) comparing the porous microplate with flask culture. Error bars, standard deviation of triplicates. **c** Cell abundance at center (*n* = 3) and surrounding (*n* = 18) wells after incubation. Asterisks denote statistical differences with following levels (two-tailed *t*-test): ***P < 0.001, **P < 0.01, *P < 0.05 and n.s. not significant.

Growth of *P. tricornutum* was measured by counting cells using a hemocytometer (Electron Microscopy Sciences) or flow cytometry (described later). Specific growth rates were calculated from the natural log of the cell densities in triplicate sampled during an exponential growth phase (day 3 for the batch culture, day 5 for the porous microplate system; Fig. 3a).

### Algal-bacterial isolates experiment

*Marinobacter* sp. 3-2 and *Algoriphagus* sp. ARW1R1 were prepared by inoculating a single colony into marine broth and growing overnight (30 °C, 150 r.p.m.). The cells were washed with f/2-Si twice by centrifuging at 2258 rcf for 4 min and diluted to a density of ~4 × 10^6^ cells ml^−1^ (*Marinobacter* sp. 3-2) and ~4 × 10^5^ cells ml^−1^ (*Algoriphagus* sp. ARW1R1). The cell densities were initially set to resemble in situ conditions with *P. tricornutum*-associated bacterial communities where *Marinobacter* displayed relative abundances several fold higher than *Algoriphagus* (Supplementary Note S1) [16]. The diluted bacterial cultures were transferred to the surrounding wells of the experimental microplate with pre-acclimated *P. tricornutum* with a density of ~1 × 10^7^ cells ml^−1^ in the center well, as described above (Fig. 1c).

Every 2–3 days, 5 µl samples were collected from wells and transferred to a 96-well plate (Corning). Before collecting the samples, the culture was thoroughly mixed using a micropipette to avoid sampling bias resulting from any spatial heterogeneity. Cells were then diluted 35 times with 1 x phosphate-buffered saline solution (PBS) (Mallinckrodt), followed by the addition of 16% formaldehyde (Thermo Scientific) to a final concentration of 2% adjusting to a final volume of 200 µl. Fixed samples were stored at 4 °C for no more than 4 weeks. After collecting the cell samples for 20 days, the remaining cultures were taken out from the device and were streaked on marine broth agar plates to check if any of the culture wells had been cross-contaminated, based on different colonies morphologies (Supplementary Fig. S3). We detected cross-contamination in 5 out of 108 bacterial samples, and they were excluded from the analysis. No less than three replicates were retained after excluding the cross-contaminated samples.

### Flow cytometry

Fluorescent counting beads for flow cytometry (Alignflow^TM^, Thermofisher) were diluted 20 times with PBS and 50 µl was added to each well of the 96-well plate for calibration. SYBR Green I nucleic acid stain (Thermofisher) was added to the samples with a final concentration of 0.1% v/v, and allowed to sit at room temperature at least for 30 min without light exposure.

Flow cytometry analysis was conducted on a BD FACS Canto II HTS to quantify the number of algal and bacterial cells with parameter setting as follows: (voltage) FSC = 580, SSC = 370, GFP = 400, PE = 330, PerCP = 647, PE-Cy7 = 677, Alexa Fluor 680 = 290, APC-Cy7 = 410, Pacific Blue = 440, AmCyan = 539. Populations were plotted with GFP-A and APC-Cy7-A intensities, allowing a clear distinction between counting beads, stained algal, and bacterial cells. The data were exported to.csv files using FlowJo (BD) and converted into cell density data using MATLAB (Mathworks).

### Bacterial community experiment

To examine bacterial community structure changes induced by the presence of the diatom, ~1 × 10^6^ *P. tricornutum* cells ml^−1^ were placed in the center well and the results were compared to control incubations where sterile media was placed in the center (Fig. 1d). Phycosphere enrichment samples [11, 16] (with the algal cells removed with a 0.8 μm filter) were used as a bacterial community inoculant where 100 µl samples were placed in the surrounding wells in the array. Layer 1 and Layer 2 refer to bacterial wells with a distance from the centre well of 8 mm and 16 mm, respectively. On the outmost well, 100 µl sterile f/2-Si medium was inoculated as a blank control for 16 S rRNA community analysis.

The devices were incubated in a container (GasPak™ EZ container systems, BD) which was filled with f/2-Si medium. All preparation steps were performed in a laminar flow hood to prevent any bacterial contamination. The container was incubated under the same condition as described above. After 7 days, each well sample was collected and filtered into 96-well filter plate (0.2 μm pore size, Pall AcroPrep) to remove liquid, and was stored at −20 °C until further analysis.

### 16 S ribosomal RNA gene amplification and sequencing

Twenty microliters of sterile DNA-free water were added to each well of the filter plates, and cells were lysed to release the DNA with heat (95 °C for 10 min). The 16 S rRNA gene was directly amplified without cleanup, using primers targeting the v4 region and modified to include Illumina platform adaptor sequence on the 5' ends. The PCR contained 10 μl of 5 Prime MasterMix, 1 μl of 10 μM of forward 16 S primer (5' - TCGTCGGCAGCGTCAGATGTGTATAAGAGACAG[GTGYCAGCMGCCGCGGTAA] − 3') [34], 1 µl of reverse 16 S primer (5' - GTCTCGTGGGCTCGGAGATGTGTATAAGAGACAG[GGACTACNVGGGTWTCTAAT] -3') [35], and 5 μl of DNA template (actual primer sequences highlighted with brackets). Cycling conditions were as follows: denaturation at 94 °C for 3 min, followed by 30 cycles of denaturation at 94 °C for 45 s, annealing at 51 °C for 30 s and extension at 72 °C for 1.0 min. The final extension was conducted at 72 °C for 10 min and the samples were held at 4 °C. The second round of amplification added Illumina Dual Nextera XT indexes and sequencing adaptors [36]. Each library was quantified with a Qubit broad range dsDNA assay and equimolar amounts of each library were pooled. The size and concentration of the final pool were verified by a D5000 High Sensitivity assay on the Agilent Tapestation. Six pM of the pooled libraries were combined with 15% phiX and paired-end sequenced on an Illumina MiSeq for 500 cycles.

Paired-end MiSeq reads were filtered to remove read pairs that contained Illumina adapter or barcode kmers with bbduk v38.22 using options *k* = 31 and hdist = 1 [37]. Read trimming and quality filtering was done with DADA2 v1.12.1 using options trimLeft = c(4, 14), truncLen = c(200,150), maxN = 0, maxEE = c(2, 2), truncQ = 2 [38]. Read pairs remaining after filtering were further processed into amplicon sequence variants (ASVs) retaining ASVs of length 274 nucleotides with at least 2 reads in at least 2 samples. The sequences were then aligned with MUSCLE v3.8.1551 [39] and clustered into an approximately-maximum-likelihood tree with Fasttree v2.1.10 [40]. Taxonomy was assigned using IDTAXA [41] against the Silva version 132 SSU database [42]. ASVs were analyzed using the R package phyloseq v1.30.0 [43]. Samples were quality-filtered by removing taxa not found at least 10 times in at least 3 samples and normalized by Cumulative Sum Scaling (CSS) method using the R package metagenomeSeq v1.28.2 [44]. In brief, a mean of 95,770 (from 11,535 to 241,656) and 1477 (from 61 to 5183) read pairs were sequenced per sample of bacterial wells and of quality-filtered negative controls, respectively (Supplementary Table S2). Quality-filtered negative samples have been selected from 18 total negative samples based on their read pairs of less than 10,000. The other five negative control samples with reading pairs of more than 10,000, were dropped from the analysis as they suggested that cross-contamination had occurred from the adjacent bacterial communities for these samples. Another 36 bacterial cultures, located in between layers 1 and 2, were not assigned with a layer number and excluded from the statistical analysis. R package phyloseq v1.30.0 was used to perform principal coordinate analysis (PCoA), vegan v2.5.6 to perform permutational multivariate analysis of variance (PERMANOVA) [45] based on weighted Unifrac [46], and R function (aov) was used to perform one-way and two-way analysis of variance (ANOVA) tests using packages tidyverse v1.3.0 [47], vegan v2.5.6 [48].

## Results

### *P. tricornutum* accumulates in a porous microplate

HEMA–EDMA is a nanoporous copolymer and can easily dry out in standard laboratory conditions (Fig. 2b, c). To circumvent the dehydrating issue, the porous microplates were immersed in f/2-Si medium in a single-well plate with a lid where ~95% of the initial 100 µl volume was retained over two weeks (Supplementary Table S3). We measured cell abundances of *P. tricornutum* incubated in the center well of the microplate and compared them with conventional batch cultures (Fig. 3a). Unexpectedly, *P. tricornutum* in the porous microplate reached a density of (2.3 ± 0.8) × 10^8^ cells ml^−1^, which was ~20 fold higher than those under a batch condition (Fig. 3b). However, the two culture methods did not show a difference in the growth rate, suggesting the copolymer did not impact *P. tricornutum* physiology once it was acclimated to the environment (*P* = 0.413, Fig. 3b inset). A rapid drop in cell densities was also observed at the beginning of the microplate incubation (day 0–2, Fig. 3b), presumably due to the lower pKa of the monomer hydroxyethyl methacrylate (HEMA) than the pH of f/2-Si medium [49], which likely impacted cell physiology initially.

We hypothesized that the higher carrying capacity in the microplate might be caused by the f/2-Si medium nutrients diffusing into the well as the algae grew, since nutrients are constantly consumed and a concentration gradient of nutrients would be created across the porous HEMA–EDMA [30]. We believe nutrient transport by fluid flow across the porous structure can be neglected, as the rate is small compared to the gradient-induced diffusion (Supplementary Note S2). We also believe vapor transport from surrounding culture wells can be neglected, as we did not observe any notable replenishment of the well volumes, and the nutrients (nitrate, phosphate) have low volatility. To test our hypothesis on the nutrient diffusion by the concentration gradient, we designed a porous microplate of a well array, one center and six outer wells, so that nutrients outside the microplate can supplement the outer wells faster than the inner wells (Fig. 3a). Indeed, algal populations cultured in the outer wells reached higher concentrations than cultures in the center (*P* < 0.01, Fig. 3c), but those at both locations still exhibited higher abundances than standard batch cultures. Growth rates in the surrounding wells were also statistically higher, 0.25 ± 0.04 d^−1^, compared to the center wells, 0.18 ± 0.03 d^−1^ (*P* < 0.05, two-tailed *t*-test). One further experiment confirmed that the inorganic nutrients in the outer reservoir diffused into the culture wells; we compared *P. tricornutum* abundances in unaltered medium with post-experiment reservoir medium, testing if the spent medium had lower nutrient concentrations. As expected, *P. tricornutum* grew slower and to lower concentrations in this spent medium compared to fresh medium, consistent with the hypothesis of inorganic nutrient diffusion from the reservoir into the wells (Supplementary Fig. S4).

### Predictive modelling of nutrient concentrations around an alga and in the porous microplate

**Fig. 4:**
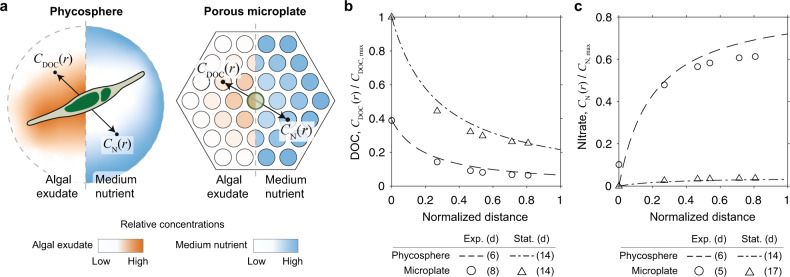
Numerical estimation of nutrient concentrations. **a** Schematic of two contrasting concentration gradients of algal dissolved organic carbon (DOC) and medium nutrients around a cell and in the porous microplate with *P*. *tricornutum* at the center and without bacteria. Numerical simulation of (**b**) DOC and (**c**) nitrate concentrations comparing the phycosphere (*Synechococcus bacillaris*) and the porous microplate at exponential (exp.) and stationary (stat.) growth phases. Nutrient concentrations and distances were normalized by their maximal values (Supplementary Note S3). Parameters for estimating phycosphere concentrations were based on data previously collected in a separate study [50].

After observing *P*. *tricornutum* drawdown of inorganic nutrients in the wells of the porous microplate, we further designed it to build a testable analogous model to the microscale processes that occur at the single cell level in the algal phycosphere [24]. At the scale of a single alga, its photosynthetic activity creates two contrasting concentration gradients governed by the laws of diffusion: an increase of algal exudates and a decrease of inorganic nutrients towards the cell. Similarly, the location of the porous microplate wells at different distances from the algal culture can artificially create distinct zones comprised of different concentrations of both algal-derived metabolites and medium inorganic nutrients, although it does not specifically test algal-attached bacteria (Fig. 4a).

To determine how the porous microplate system can mimic the phycosphere environment from the point of view of algal-associated bacteria, we constructed a numerical model comparing spatiotemporal profiles of nutrients and exudates at these two different scales. For the phycosphere model, we tested four taxonomically diverse phytoplankton species, using published data from laboratory cultures [50]. This allowed us to infer spatial concentrations of dissolved organic carbon (DOC) and nitrate, as proxies of algal exudates and inorganic nutrients respectively, to directly compare to the modeled profiles in the porous microplate system with *P*. *tricornutum* (Supplementary Note S3). Regardless of growth phase, we found algal released compounds decrease in concentration by 3–7 fold with increasing distance from the cell surface, supporting the theoretical validity of the phycosphere [1, 24] (Fig. 4b, Supplementary Fig. S5). However, inorganic nutrients are rapidly consumed by the cells while they grow [50, 51], and the concentration of nitrate becomes low as observed in the numerical model when the culture reaches stationary phase (Fig. 4c, Supplementary Fig. S5). Similar results were obtained in the simulated porous microplate system, with a ~5 fold decrease in DOC concentration with increasing distance from the center alga-located well as well as the depletion of nitrate in the center well within 6 days (Fig. 4b, c).

### Bacteria respond to gradients of *P. tricornutum* exudate and medium nutrients

**Fig. 5:**
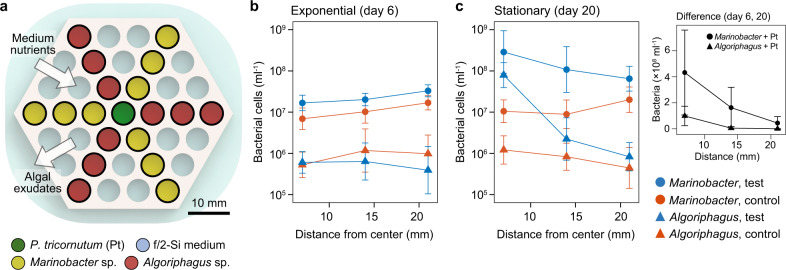
Measurements of bacterial growth in the porous microplate. **a** Schematic of a microplate depicting well locations of *P. tricornutum* and two bacterial strains, *Marinobacter* sp. 3-2 and *Algoriphagus* sp. ARW1R1. Abundance of the two bacterial strains sampled at (**b**) exponential and (**c**) stationary phase co-cultured with *P. tricornutum* (test) or without (control); (inset) difference of the abundance between the timepoints. Error bars, geometric standard deviations [64] of the following number of replicates: *n* = 5 (*Algoriphagus* with *P. tricornutum*, distance 21 mm), *n* = 8 (*Algoriphagus* without *P. tricornutum*, distance 21 mm), *n* = 9 (all others).

Following the experimental and numerical results on nutrient availability around the algal host, we next explored how algal-associated bacteria grow and respond to the diffusion of algal and medium nutrients in the porous microplate. In an array of microplate wells, *P. tricornutum* and two bacterial strains, *Marinobacter* sp. 3-2 and *Algoriphagus* sp. ARW1R1, were co-cultured at different distances from one another (Fig. 5a). The cells were routinely sampled to measure their abundances for three weeks, a period that did not limit algal growth as the surrounding f/2-Si medium is rich with inorganic nutrients. The two bacterial strains were previously isolated from *P. tricornutum* enrichments [16] and are relatively abundant in laboratory cultures where bacterial growth depends on *P. tricornutum* photosynthate [11]. The abundances of the two strains at different time points were compared to one another as a function of the distance away from the center well, which contained either *P. tricornutum* (“test”) or f/2-Si medium (“control”).

During the initial algal growth phase (day 0–6), we observed a rapid increase of bacterial abundances (Supplementary Fig. S6). Interestingly, *Marinobacter* was more abundant as distance from the center well increased, regardless of the presence of *P. tricornutum* (*P* < 0.05, Fig. 5b). However, *Algoriphagus* did not exhibit any such difference (*P* = 0.814 with algae and *P* = 0.165 without). This tendency of higher abundances in the outer wells was also observed for *Marinobacter* without *P. tricornutum* at the other timepoints in the early growth phase (day 2–8, *P* < 0.05, Supplementary Fig. S6).

We expect that medium inorganic nutrients were consumed by algal photosynthesis, as shown in our previous experiments, creating a concentration gradient of these inorganic nutrients increasing towards the outer microplate wells (Supplementary Fig. S7). This suggests that *Marinobacter* sp. 3-2 growth depended on the inorganic nutrients as well as algal DOC at this early growth phase. For the treatment without *P. tricornutum* where we also detected increasing growth towards the outer wells, there was likely also a gradient in inorganic nutrient concentration. This is because the outer wells are adjacent to the medium input outside the microplate, whereas the inner wells are in the center and adjacent to other wells with potentially competing bacteria. Nonetheless, *Marinobacter* co-cultured with algae were more abundant than without (*P* < 0.001, Fig. 5b), suggesting that the bacteria also used some metabolites from *P. tricornutum* for growth and that these metabolites were able to diffuse all the way to the outermost layer.

Results from later in the growth phase (e.g., day 17–20) were drastically different, where the two bacterial strains exhibited distinct responses to the spatial arrangement. On day 20, the abundances of both strains strongly increased with proximity to *P. tricornutum*, indicating that their growth was correlated to algal DOC diffusion (*P* < 0.001, Fig. 5c). *Algoriphagus* appeared to have a greater dependence on *P. tricornutum* exudates than *Marinobacter*, as measured by a stronger decrease in its abundance with increasing distance from the center (*Algoriphagus*, 97 fold; *Marinobacter*, 6 fold). Furthermore, in the outermost well, *Marinobacter* was 100 times more abundant than *Algoriphagus*, suggesting that *Marinobacter* does not require spatial proximity to *P. tricornutum* for its growth. Even noting that initial *Marinobacter* abundance was ten times greater than *Algoriphagus*, this still implies that *Marinobacter* has an ability to utilize f/2-Si nutrients for its growth while *Algoriphagus* does not. Indeed, the growth of *Algoriphagus* located further away from *P. tricornutum* (in the outer wells) was negligible over the 20 days of incubation period, whereas *Marinobacter* grew in all the wells (Supplementary Fig. S6). The innermost well was the only location where *Algoriphagus* grew, suggesting the algal DOC needed by *Algoriphagus* did not diffuse further, or was fully incorporated by *Algoriphagus* cells in the innermost well.

### Spatial influence on bacterial community development

**Fig. 6:**
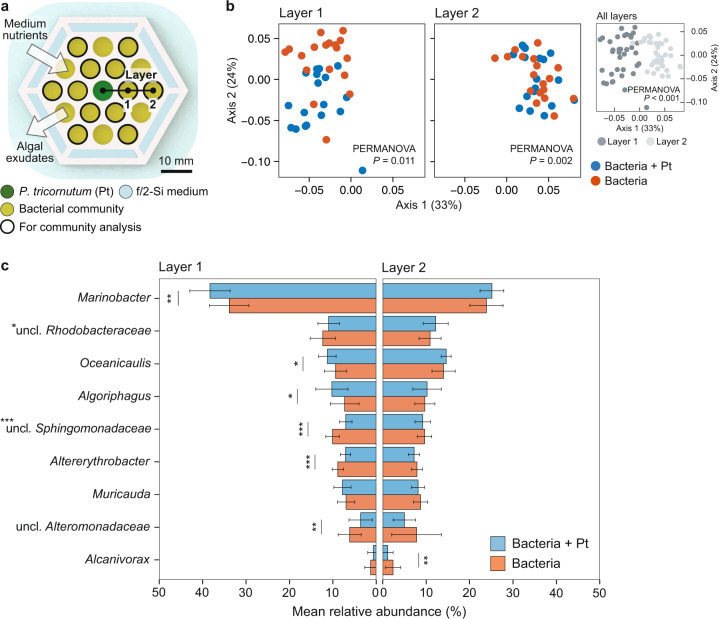
Bacterial community analysis in the porous microplate. **a** Schematic of a microplate depicting locations of *P. tricornutum* and associated bacterial communities. **b** Principal coordinate analysis of the communities using weighted Unifrac metric [46] with *P*-values (PERMANOVA) on structural difference between layer conditions (inset) and the two treatments. **c** Genus level taxonomic abundance of the communities comparing two treatments incubated for 7 days (means more than 0.5% displayed). Error bars, standard deviations of 18 replicates. Asterisks next to genera (y-labels), statistically significant interaction term of layer number and treatment (two-way ANOVA). Asterisks next to color bars, statistical significance of differences between two treatments (one-way ANOVA) with following levels: ***P < 0.001, **P < 0.01, *P < 0.05.

Our previous experiment documents the differential response of two bacterial strains grown in monoculture exchanging metabolites with the algal host and potentially with one another. The next step was to test our system with mixed communities and examine how community development is influenced by distance from the alga. By inoculating *P. tricornutum* in the center well and by filling the container with f/2-Si medium outside (Fig. 6a), we expect that a spatial gradient of increasing inorganic nutrients (higher outside, lower inside) and an opposite gradient of algal DOC (higher inside, lower outside) is generated along the well array, similar to gradients that are hypothesized to exist in the phycosphere at the single cell level. The algal and bacterial cells were incubated in the microplate for a week, a period long enough to elicit population level changes in previous experiments, as discussed above. Bacterial communities were then analyzed by sequencing their 16 S ribosomal RNA (rRNA) genes, enabling us to examine how community development is affected by physical location along the two gradients.

For a global analysis, samples were grouped by treatment (with or without *P. tricornutum* in the middle well) and layer number (layer 1 located at the inner and layer 2 at the outer well). Data visualized using principal coordinate analysis (PCoA) showed a notable difference between layer 1 and layer 2 regardless of the treatment (*P* < 0.001, Fig. 6b inset), suggesting the community structure was influenced by f/2-Si nutrient availability. In both layers, the sample groups were statistically different between treatments (*P* < 0.05), but the structural difference was most visibly obvious in layer 1 compared with layer 2 with a higher proportion of the variance explained by the principal coordinates (Fig. 6b, Supplementary Fig. S8). This suggests that the community structure was indeed affected by algal photosynthetic activity and exudate production, and to a greater extent for bacterial populations located closest to *P. tricornutum*. Across all 72 community samples, nine ASVs were ubiquitous (found in all samples), assigned to the genera *Muricauda*, *Marinobacter*, *Oceanicaulis*, *Altererythrobacter* and the families *Rhodobacteraceae* and *Sphingomonadaceae* (details provided in Supplementary Fig. S9).

To investigate in greater detail which taxa were influenced by spatial proximity to the algal cells, relative abundances were quantified by merging amplicon sequence variant (ASV) reads at the genus level and compared between well location (inner versus outer) and treatment (with and without algae in the center well). Comparing between treatments (with or without algae), the communities incubated closest to the center well (layer 1) contained more genera that were statistically different in their abundance than in layer 2 (Fig. 6c). In detail, six genera, *Marinobacter*, *Oceanicaulis*, *Algoriphagus*, unclassified *Sphingomonadaceae*, *Altererythrobacter* and unclassified *Alteromonadaceae*, were different in layer 1, whereas only *Alcanivorax* showed such difference in layer 2 (*P* < 0.05). We also observed an interaction between treatment and well location with genera *Marinobacter*, unclassified *Rhodobacteraceae*, unclassified *Sphingomonadaceae*, *Muricauda* and *Altererythrobacter*, suggesting that they were affected by physical proximity to *P. tricornutum* in the center well and inorganic nutrients on the outside of the microplate (*P* < 0.05). Specifically, the genera *Marinobacter*, *Oceanicaulis*, *Algoriphagus*, and *Muricauda* exhibited higher relative abundances in co-cultures with algae compared to without, suggesting that their response to the algal exudate was stronger than their response to inorganic nutrients. On the other hand, the genera unclassified *Rhodobacteraceae*, unclassified *Sphingomonadaceae*, *Altererythrobacter* and unclassified *Alteromonadaceae* were relatively less abundant with algae compared to without, indicating they were more strongly influenced by inorganic nutrients, or more likely did not respond to algal exudates as well as the other taxa.

## Discussion

One major unanswered question in the study of algal-bacterial interactions is how the spatial and temporal dynamics of algal exudation and inorganic nutrient uptake contribute to microbial community development around the phycosphere [11, 52]. Designing experiments to answer such questions with standard laboratory cultures has been particularly difficult, as no method exists to isolate the contributions of these components. We shed some insight on the problem by using spatial arrays of culture wells with a porous microplate and studied how bacteria grew under different nutritional levels of algal exudates and inorganic compounds. After analyzing bacterial responses both as single isolates and at the mixed community level, we found evidence that certain taxa became more abundant towards algal-derived organic matter, while others responded more strongly to inorganic nutrients away from the algal cells. The latter finding is unexpected, particularly since the initial bacterial communities originated from algal co-cultures where the only source of organic carbon was algal photosynthesis.

Our findings have a major impact on our current understanding of how free-living heterotrophic bacteria respond to the microscale environment around an algal cell. The concept of the phycosphere is based on the idea that organic matter exuded by the algal cell creates a gradient of decreasing concentrations going away from the alga that specific bacteria can take advantage of, and our experimental and modeling data support this well-developed idea. Our experimental and modeling data also suggest that there is a potentially equally important gradient that has thus far been largely ignored: the gradient of inorganic nutrients, which in this case is opposite, increasing in concentration away from the algal cell. It may at first seem odd that inorganic nutrient gradients have not been investigated in the context of heterotrophic bacterial community structure, despite the known ability of bacteria to consume inorganic compounds such as nitrate [51, 53–56], phosphate [53, 57] and ammonium [57]. However, heterotrophic bacteria in surface waters are generally known as producers of inorganic nutrients rather than consumers [3], and since algae incorporate more of those inorganic nutrients than heterotrophic bacteria (due to their larger sizes and higher nutrient flux constants), bacteria are not thought to be affected by inorganic nutrient gradients around algal cells. Our data suggest that this assumption is incorrect, and may be particularly critical in high inorganic nutrient ecosystems, such as coastal oceans and engineered algal biofuel ponds designed to maximize the growth of algae [58]. We show that a subset of the bacterial community in these environments can benefit from both inorganic nutrients and organic exudates, suggesting that there are at least 2 different niches in the phycosphere which should support at least 2 bacterial lifestyles. The first niche supports bacteria adapted to incorporate complex algal organic matter, and these organisms only respond to the organic matter gradient and grow best closest to the algal cells and later in the bloom or culture growth cycle, when inorganic nutrients are depleted and algal exudates are more complex (and diffuse slower). This first niche is exemplified by *Algoriphagus*. The second niche, here represented by *Marinobacter*, is filled by bacteria that grow using both algal organic matter and inorganic nutrients, and these organisms are likely mutualistic in the early growth phase because there are enough inorganic nutrients for both algae and bacteria (thus no competition for inorganic nutrients). It is likely that these bacteria could become competitive under low inorganic nutrient concentrations, unless they switch their metabolisms from incorporating both organic and inorganic nutrients to incorporating only organic nutrients, a concept that remains untested as of now.

Of further note, our co-culture system illuminates how bacterial responses are governed by the combination of algal growth phase and physical distance in a taxon-specific manner. We observed *Algoriphagus* exhibiting a greater spatial dependency on *P. tricornutum* than *Marinobacter* in the isolates experiment, and these responses were confirmed in mixed populations in the community experiment. These experimental observations in the porous microplate enabled us to conceptualize a unique nutrient profile spatially determined by algal growth phase, and we were able to construct a mathematical model of nutrient concentrations around an alga (Fig. 4a), which simultaneously addresses gradients of exudates and inorganic nutrients under different laboratory growth phases. This model allows us to estimate how the algal microbiome might respond to the spatial distribution of the two nutritional factors (algal exudates and inorganic nutrients), because we expect certain bacteria to reproduce at faster rates in locations where more nutrients are accessible. Specifically for the exponential algal growth stage, we expect certain bacteria to exhibit a faster growth rate outside of the phycosphere (i.e. free-living), because there are still inorganic nutrients available in the bulk medium and smaller molecules exuded from the algal cells can diffuse further away. On the other hand, for algal cultures in stationary phase, other bacteria tend to attach to algal cells or stay within the phycosphere, since inorganic nutrients are depleted throughout the medium and larger molecules diffuse slowly from the surface of the algal cells. Indeed, this scenario explains some experimental observations where the number of attached bacteria increased as algal cultures aged [13, 59].

It should be noted that our proposed system does not address responses of bacteria that physically attach to algae, which is another major contribution to phycosphere interactions [24]. Physical attachment allows bacteria to use high molecular weight carbon compounds that are bound to the surface such as polysaccharides [60], but they have negligible diffusivity in water and in the porous copolymer HEMA-EDMA. To circumvent this limitation, in the single isolates experiment we have selected model organisms that do not attach or rarely attach to *P. tricornutum* even though the organisms were isolated from a “phycosphere enrichment” community [11, 16]. Nonetheless, the design does not explain whether other algal-attaching bacteria will respond and how they impact host physiology, which is important but remains unanswered.

Notably, by adopting the porous microplate system we constructed a unique environment for algal cells distinct from standard batch cultures, where in the microplate nutrients constantly diffused into the growing culture from outside through the nanoporous copolymer. A supply of fresh nutrients into an algal culture is often found in (semi-) continuous systems; however, our system is also distinct from such methods since algae are not removed from the culture and they accumulate to a high cell density. Indeed, *P. tricornutum* reached abundances significantly higher than previously reported values, including under (semi-) continuous systems [61, 62]. We believe this is the first demonstration that algae can accumulate to such high densities, purely driven by a novel cultivation technique. If appropriately scaled up, this new cultivation approach could lead to more efficient algal biomass production.

Overall, the combination of our experimental culture system and a simple microscale diffusion model successfully explained the temporal and spatial association patterns between algae and different algal-associated bacteria, providing a mechanistic understanding of the distinct impact of inorganic nutrients as well as algal exudates on bacterial growth. We expect this approach can be used in future studies to investigate longstanding questions about community level interactions mediated by metabolite exchange at different spatial scales, including testing hypotheses about bacteria-phycosphere interactions [11, 13, 16], but also more general microbiome interactions in soil, plant, and animal models.

## Supplementary information

Supplementary information
