# Supplementary material for: Bacterial response to spatial gradients of algal-derived nutrients in a porous microplate
Source: ISME J. 2021 Nov 17;16(4):1036–45. doi: 10.1038/s41396-021-01147-x (PMC8940921; doi:10.1038/s41396-021-01147-x)
Supplement: Supplementary file 1 — Supplementary information [file 41396_2021_1147_MOESM1_ESM.docx]

**Supplementary Information**

Bacterial Response to Spatial Gradients of Algal-Derived Nutrients in a Porous Microplate

Hyungseok Kim,^1,2^ Jeffrey A. Kimbrel,^3^ Christopher A. Vaiana,^1,4^ Jessica R. Wollard,^3^ Xavier Mayali,^3^* and Cullen R. Buie^1^*

^1^ Department of Mechanical Engineering, Massachusetts Institute of Technology, Cambridge, MA, USA.
^2^ Institute for Data, Systems, and Society, Massachusetts Institute of Technology, Cambridge, MA, USA.
^3^ Physical and Life Science Directorate, Lawrence Livermore National Laboratory, Livermore, CA, USA.
^4^ Department of Biological Engineering, Massachusetts Institute of Technology, Cambridge, MA, USA.

* Correspondence: Xavier Mayali (mayali1@llnl.gov) and Cullen R. Buie (crb@mit.edu).

List of Supplementary Information

- Supplementary Figure S1. Cell images of *P. tricornutum* culture and number of attached bacteria.
- Supplementary Figure S2. Dimension of porous microplate used in the study.
- Supplementary Figure S3. Colonial morphology of bacterial isolates.
- Supplementary Figure S4. Growth of *P. tricornutum* in porous microplate-spent medium.
- Supplementary Figure S5. Numerical analyses of nutrient concentrations in porous microplate and in phycosphere.
- Supplementary Figure S6. Growth of *Marinobacter* sp. 3-2 and *Algoriphagus* sp. ARW1R1 in the porous microplate.
- Supplementary Figure S7. Numerical concentration of nitrate in porous microplate wells.
- Supplementary Figure S8. Cumulative proportion of total variance explained by number of dimensions in principal coordinate analysis.
- Supplementary Figure S9. Number of shared amplicon sequence variants (ASVs) for community analysis.
- Supplementary Table S1. List of bacterial isolates and their accession numbers.
- Supplementary Table S2. Summary of 16S rRNA sequencing result.
- Supplementary Table S3. Dehydration measurement of porous microplate well cultures.
- Supplementary Note S1. Determination of initial cell densities in bacterial isolates experiment.
- Supplementary Note S2. Estimation of flow rate through copolymer.
- Supplementary Note S3. Numerical analyses of algal dissolved organic carbon (DOC) and medium nitrate concentrations around an alga and across porous microplate wells.

**
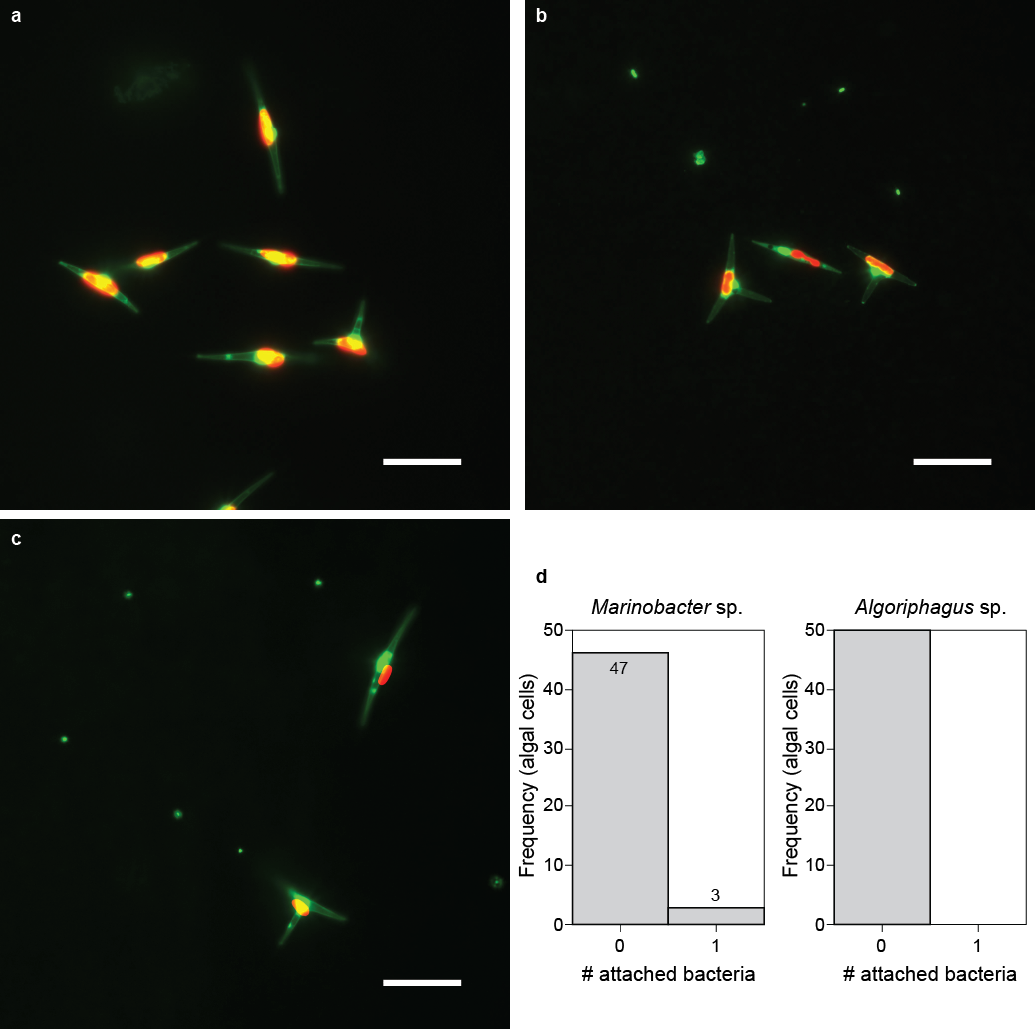
**

**Supplementary Figure S1. Cell images of *P. tricornutum* culture and number of attached bacteria.** Fluorescence microscopy images of algal cells (a) axenic, co-culture with (b) bacterium *Marinobacter* sp. 3-2 and (c) bacterium *Algoriphagus* sp. ARW1R1, displaying nucleic acid (green) and chloroplast (red). Scale bar, 20 µm. (d) Distribution of number of bacteria attached to *P. tricornutum* based on the fluorescent images with 50 single algal cells.

**Supplementary Figure S2. Dimensions of porous microplates used in the study.** Dimensions with top and bottom views of a device for (a) *P. tricornutum* incubation experiment, (b) single bacterial isolates growth experiment, and (c) bacterial community analysis experiment.

**Supplementary Figure S3. Colonial morphology of bacterial isolates.** Image of bacterial colonies (a) *Algoriphagus* sp. ARW1R1 and (b) *Marinobacter* sp. 3-2 on Marine Broth agar plate.

**Supplementary Figure S4. Growth of *P. tricornutum* in porous microplate-spent medium.** Cell numbers of *P. tricornutum* grown in spent f/2-Si medium that immerse three replicating porous microplates (spent medium), compared to the cells grown in unaltered medium of the same volume (control), no replicates.

**Supplementary Figure S5. Numerical comparison of nutrient concentrations in the porous microplate and around the phycosphere.** Nutrient concentrations and distances were normalized by their maximal values to compare between the two environments (see Supplementary Note S3 for derivation).

**Supplementary Figure S6. Growth of *Marinobacter* sp. 3-2 and *Algoriphagus* sp. ARW1R1 in the porous microplate.** Bacterial cell densities over time grown with *P. tricornutum* (red) or without (grey). Error bars, standard deviation of *n* = 9 replicates, except for the following: *Algoriphagus* layer 3 with *P. tricornutum* (*n* = 5), *Algoriphagus* layer 3 without (*n* = 8).

**Supplementary Figure S7. Numerical concentration of nitrate in porous microplate wells.** Nitrate concentrations after 5–17 days of simulated incubation with two bacterial isolates, *Marinobacter* sp., 3-2 and *Algoriphagus* sp. ARW1R1, under two treatments (a) with *P*. *tricornutum* (top row, test) and (b) without *P. tricornutum* (bottom row, control). See Supplementary Note S3 for derivation.

**Supplementary Figure S8. Cumulative proportion of total variance explained by number of dimensions in principal coordinate analysis.**

**Supplementary Figure S9. Number of shared amplicon sequence variants (ASVs) for community analysis.**

**Supplementary Table S1. Dehydration measurement of porous microplate well cultures.** Initial *P. tricornutum* culture of 100 µl was inoculated to the array of porous microplate then sampled to measure the volume after two weeks of incubation (all units in microliter).

| Location | Replicate 1 | Replicate 2 | Replicate 3 |
| --- | --- | --- | --- |
| Center | 90 | 95 | 95 |
| Surrounding 1 | 90 | 100 | 90 |
| Surrounding 2 | 100 | 95 | 90 |
| Surrounding 3 | 100 | 100 | 95 |
| Surrounding 4 | 100 | 90 | 90 |
| Surrounding 5 | 90 | 90 | 95 |
| Surrounding 6 | 100 | 95 | 95 |
| **Total mean** | **94.52** | | |

**Supplementary Table S2. List of bacterial isolates and their accession numbers.**

| Genus | Strain | IMG genome ID | Source |
| --- | --- | --- | --- |
| *Marinobacter* | 3-2 | 2785510723 | Samo *et al*. [1] |
| *Algoriphagus* | ARW1R1 | 2747842515 | Samo *et al*. [1] |

**Supplementary Table S3. Summary of 16S rRNA sequencing result.**

| Type | Number of  samples | Mean reads | Standard deviation | Minimum reads | Maximum reads |
| --- | --- | --- | --- | --- | --- |
| Negative control, total | 18 | 19,997 | 42,375 | 61 | 159,617 |
| Negative control, quality-filtered | 13 | 1,477 | 1,650 | 61 | 5,183 |
| Community without  *P. tricornutum*, total | 54 | 86,467 | 32,934 | 30,487 | 201,701 |
| Community without  *P. tricornutum*, layer 1 | 18 | 87,257 | 21,926 | 52,004 | 128,587 |
| Community without  *P. tricornutum*, layer 2 | 18 | 79,366 | 34,263 | 30,487 | 141,337 |
| Community with  *P. tricornutum*, total | 54 | 105,074 | 49,143 | 11,535 | 241,656 |
| Community without  *P. tricornutum*, layer 1 | 18 | 96,924 | 45,143 | 11,535 | 241,656 |
| Community without  *P. tricornutum*, layer 2 | 18 | 132,299 | 48,098 | 35,574 | 214,029 |

**Supplementary Note S1. Determination of initial cell densities in bacterial isolates experiment.**

In the bacterial isolates growth experiment, we tried to replicate *in situ* abundances of algal-associated bacteria naturally present in the community. To obtain the quantitative measures on the two bacterial model strains used in the experiment, *Algoriphagus* sp. ARW1R1 and *Marinobacter* sp. 3-2, we first referred to relative abundances of these bacteria given from Samo *et al.* [1]. By comparing the relative abundances from 16S rRNA gene sequencing, we determined a ratio between *Algoriphagus* and *Marinobacter* in the *Phaodactylum*-associated bacterial community sample. As shown in the table by Samo *et al*. (Appendix S2), among a total of 35 community samples of *P. tricornutum* phycosphere enrichments, *Marinobacter* was present in 34 replicates whereas only 8 contained *Algoriphagus*. For community samples that contained both genera, the abundance ratio (*Marinobacter* : *Algoriphagus*) varied from 0.1 to 18, with 1.25-fold difference in their geometric means. Considering that *Algoriphagus* was absent in most other community samples, we set out the initial bacterial cell density of *Marinobacter* to be 10-fold higher than *Algoriphagus*.

To determine the absolute cell density of the bacteria for inoculating into the porous microplate, we referred to the previously reported cell densities of *P. tricornutum* and its associated bacteria, where in most cases the two densities are comparable (for example see Diner *et al*. [2]). Because in our design with the porous microplate algal cells are located only at the center well, we set the initial algal density to have several folds higher than bacterial density, so that sufficient amount of algal metabolites diffuse towards surrounding bacterial culture wells. Given these considerations, we inoculated ~1 × 10^7^ cells
ml^-1^ of *P. tricornutum* into our porous microplate and set the initial bacterial densities to have ~4 × 10^5^ cells ml^-1^ for *Algoriphagus* and ~4 × 10^6^ cells ml^-1^ for *Marinobacter*.

It should be noted that the measures of bacterial relative abundances in Samo *et al*. are the number of 16S rRNA gene copies, different than the direct abundance of cells measured in our study. Although many factors affect the correlation between gene copies and cell densities (e.g. efficiency in cell lysis / PCR, differential 16S rRNA gene copy numbers across bacterial taxa), these data still provide the best estimate of the ratio between the two taxa.

**Supplementary Note S2. Estimation of flow rate through copolymer.**

To calculate a flow rate between a microplate well and a hydrating medium, we consider the flow across the porous structure driven by a liquid height level difference (i.e. pressure difference). We model the flow by using Darcy’s law which allows us to write the flow rate *Q* as

, (S2.1)

where *k* denotes the permeability, *A* the cross-sectional area, *µ* the viscosity, *L* the thickness of the porous structure, and *∆P* is the pressure difference across the structure.

To obtain a numerical value of *Q*, we also estimate *∆P* based on the difference between water height level, expressing it by (with density 𝜌, gravitational acceleration *g*, and height difference *∆h*). Here we note that a maximal *Q* can be obtained by finding a maximum of *∆h*, a situation where the culture volume is running low after a repeated sampling procedure in bacterial isolates experiment.

In detail, the lowest culture volume in the experiment is ~35 µl assuming no well evaporation (initial volume 75 µl with eight times of sampling, each of 5 µl), giving the culture height ~1.5 mm. The surrounding f/2-Si medium volume is 25 ml, assuming without evaporation, thereby giving the height ~5.1 mm (surface area 49.2 mm^2^, see dimensions in Supplementary Fig. S2). With the height difference ~3 mm across the porous wall, we calculate the pressure difference ~30 Pa.

Based on the dimension of a microplate, we approximate *A* as 60 mm^2^ and *L* as 1 mm. For the permeability *k* of co-polymer HEMA–EDMA, we use a previously reported value, ~8 × 10^-21^ m^2^ [3]. Finally with the water viscosity ~10-3 Pa ∙ s, we obtain a maximal flow rate ~10^-8^ µl/s ≈ 10^-3^ µl/d. Considering a microplate well has a volume ~100 µl, we conclude that the liquid flow is negligible to our experimental incubation period.

**Supplementary Note S3. Numerical analyses of nutrient concentrations in the porous microplate and in the phycosphere.**

We hypothesize that the algal growth phase in the laboratory cultures determines the spatial gradient of algal exudates and inorganic nutrients. As the cells senesce, the average molecular weight of released compounds increases from low [4–7] to high [4, 7–9], meaning the exudate compounds diffuse at slower rates as the cultures age. On the other hand, inorganic nutrients are constantly consumed throughout culture growth so the overall amount will decrease accordingly. This allows us to model the spatial concentration of algal exudates and inorganic nutrients at different growth phases at the scale of a single alga and the scale of a porous microplate. As a proxy for all inorganic nutrients, nitrate was chosen as it is the inorganic nitrogen source used in our experiments and is expected to show similar diffusivities to other medium components (phosphate, trace metals, etc). As a proxy for algal exudates, dissolved organic carbon was selected as it is the only source of bacterial remineralization.

*Modeling nutrient concentrations around an alga (phycosphere)*. We first begin with Fick’s second law of diffusion, , where *C* is the concentration of a nutrient and *D* is the diffusion coefficient. Here we exclude the contribution of fluid advection, because a cell size of ~10 μm with our model organism *P. tricornutum*, results in a small Peclet number ranging from 0.01 to 0.1 [4]. We assume an alga as a sphere of a radius *r*_0_, exuding DOC at a rate *Q*_DOC_, and consuming nitrate at a rate *Q*_N_ (e.g. mol/s). Under a quasi-steady state where the transient term of the diffusion equation is negligible, we write the concentration of DOC and nitrate as

, (S3.1)

, (S3.2)

where *r* is radius from the cell center, *C*_inf_ is nitrate concentration far away from the cell, and *D*_DOC_, *D*_N_ are diffusion coefficients of DOC and nitrate respectively [4, 10].

In order to compute *C*_DOC_ (*r*) and *C*_N_ (*r*), we consider a range of domain *r* with algal cells equally distributed with density *ρ*. Because the average distance between cells is ~2(3 / 4π𝜌)^1/3^, we denote *R* by half of the distance, (3 / 4π𝜌)^1/3^, and let *r* range from *r*_0_ (surface of a cell) to *R* (equal distance between two adjacent cells). Noting that nitrate has a relatively low molecular weight whereas DOC compounds can depend on an algal growth phase [4–9], the diffusion coefficients are approximated as *D*_N_ = 10^-9^ m^2^/s, *D*_DOC_ = 10^-9^­ – 10^-10^ m^2^/s (from exponential to stationary phase).

We note that DOC and nitrate concentrations experimentally measured at *i* th sampling timepoint (day), respectively, denoted by and , can represent spatial average concentrations around the cell. Therefore we write and , where *V* is the volume for which the concentration is spatially averaged within the domain *r* ∈ (*r*_0_, *R*). Following Equations
(S3.1-2), this allows us to write

, (S3.3)

, (S3.4)
where physical quantities with upper index *I* denote those measured (or computed) on *i* th sampling day.

Additionally, we assume at the surface of a cell that nitrate concentration is nearly zero as the nutrients are quickly consumed by algae, which has experimentally been shown with nitrate and *P. tricornutum* [2]. This allows us to construct a boundary condition at *r* = *r*_0_ of the nitrate concentration, giving

. (S3.5)

We estimated values of , , by solving Equations (S3.3-5) and obtained nutrient concentration profiles with Equations (S3.1-2). Numerical calculations were performed using MATLAB (Mathworks) based on the measurements on average DOC and nitrate concentrations previously reported by Biddanda *et al.* [7]. To properly compare the results to under a microplate system, the DOC and nitrate concentrations were normalized by *C*_DOC_ (*r*_0_) at day 14 (denoted as *C*_DOC,max_) and *C*_N_ at day 0 (denoted as *C*_N,max_), respectively. Distance *r* was also rescaled to write (*r* – *r*_0_) / (*r* – *R*), the normalized distance from the surface of a cell. For displaying Figure 4b,c, *Synechococcus bacillaris* was chosen among the four phytoplankton species, because its cell densities were the most similar to those of *P. tricornutum*. For displaying Supplementary Figure S5, measurements of all four model species were used to estimate the nutrient concentrations.

*Modeling nutrient concentrations in porous microplate wells*. For estimating nutrient concentration in the wells of porous microplate, we model each culture well (and surrounding f/2-Si medium) as a node of a network, where adjacent wells exchange the nutrients based on the diffusion law. In specific, the mass conservation equation allows to express *C_i_* (*t*), the nutrient concentration at time *t* at node *i*, by

$\frac{d}{dt}\left(C_{i}\cdot V\right)=-R_{0i}+\sum_{j=1}^{N} J_{ji}\cdot A_{ji}$, (S3.6)

where *V* is the culture volume, *R*_0_*_i_* the nutrient consumption rate by the culture, *J_ji_* the flux from node *j* to *i*, and *A_ji_* the characteristic cross-sectional area between the node *j* and *i*, and *N* is the total number of microplate wells and the surrounding medium. We expand *J_ji_* by using Fick’s first law of diffusion,
*J_ji_* = *D* (*C_j_* – *C_i_*) / *l_ji_* , with diffusion coefficient *D* (through the copolymer), distance between adjacent wells *l_ji_*. Hence we continue to write the Equation (S3.6) as

$\frac{d}{dt}\left(C_{i}\cdot V\right)=-R_{0i}+D\sum_{j=1}^{N} \frac{A_{ji}(C_{j}-C_{i})}{l_{ji}}$. (S3.7)

We divide Equation (S3.7) with *V* to express at the unit volume, and let *R_i_* denote as the consumption rate per cell, 𝜌 the cell density, and *A*_0_ as the side area of a culture. This gives

$\frac{dC_{i}}{dt}=-\rho_{i}R_{i}+\frac{DA_{0}}{V}\sum_{j=1}^{N} \frac{A_{ji}(C_{j}-C_{i})}{A_{0}l_{ji}}$. (S3.8)

Now we discretize Equation (S3.8) in time, giving

. (S3.9)

with *d* the well diameter, 𝛥*t* time increment, and 𝜑*_ji_* the ratio between *A_ji_* and *A*_0_ (see figure). We also rewrite the conservation Equation (S3.7) with *C*_res_, the concentration of reservoir that surrounds the microplate, and *V*_res_, the volume of the reservoir (note that this is different than *V*). Similarly repeating the same analysis in Equations (S3.7-9), we obtain

, (S3.10)

which is discretized in time to write

. (S3.11)

Geometrical interpretation of the Equations (S3.9, S3.11) is given in the diagram below (Figure), allowing us to determine *A_ji_* and *l_ji_* based on the dimension of a microplate. Note that *A_ji_* and *l_ji_* are symmetric; in other words, *A_ij_* = *A_ji_* and *l_ij_* = *l_ji_* , thereby allowing to determine the values at *i* = 38 (reservoir).

Figure. Determination of characteristic area ratio 𝜑_ji_ and distance between adjacent wells *l*_ji_ for every node *i*. Well location is shown in the top left diagram, labeled with the node *i* (typed boldface). Otherwise displayed above for each case from (i) to (v), it is assumed that the wells are not adjacent; 𝜑_ji_ = 0 and *l*_ji_ = 0.

Parameter settings to solve Equations (S3.9, S3.11) are given in the table below. In specific, molecular diffusivity in nanoporous copolymer HEMA–EDMA was set to have the same order as reported in Ge *et al*. [11], with DOC three time lower than nitrate considering its higher molecular weight. Nitrate uptake rate *R*_N_,*_i_* by bacterium *Algoriphagus* (*i* = 2,4,6,8,10,12,14,16,18) and alga *Phaeodactylum* (*i* = 1) was respectively calculated from the collected data by Kirchman [12] and Diner *et al.* [2] (maximal rate selected from daily concentrations of the nitrate). Uptake rate by bacterium *Marinobacter* (*i* = 3,5,7,9,11,13,15,17,19) was determined to have a value geometrically intermediate between the rates of *Algoriphagus* and *Phaeodactylum*, as *Marinobacter* has shown its ability in the isolates growth experiment to consume the inorganic nutrients better than *Algoriphagus* yet still lower than the alga. DOC production rate by *P. tricornutum*, *R*_DOC_,*_i_*, was calculated based on the numerical model provided by Seymour *et al*. [4] and Mullin *et al*. [13]. Cell density 𝜌*_i_^t^* was obtained from the experimental measurements on days 0,2,4,6,8,11,14,17,20 and their logarithmic interpolations between the timepoints (Supplementary Figure S6). Culture volume *V _i_^t^* was initially set to have 75 µl and to decrease stepwise by 5 µl at every sampling timepoint (see Materials and Methods). Initial value of the nitrate concentration *C*_N_ was set to have 880 µM, same as in the f/2 medium.

| Parameter | Value | Note / source |
| --- | --- | --- |
| *R*_N_,*_i_* | 10^-8^ μmol/h-cell | *P. tricornutum* (*i* = 1) [2] |
| *R*_N_,*_i_* | 10^-9.5^ μmol/h-cell | *Marinobacter*  (*i* = 3,5,7,9,11,13,15,17,19) [12] |
| *R*_N_,*_i_* | 10^-11^ μmol/h-cell | *Algoriphagus*  (*i* = 2,4,6,8,10,12,14,16,18) |
| *R*_DOC_,*_i_* | –7.2 × 10^-6^ μmol/h-cell | *P. tricornutum* (*i* = 1) [4] |
| *D*_DOC_ | 10^-10^ m^2^/s | DOC diffusivity |
| *D*_N_ | 3 × 10^-10^ m^2^/s | Nitrate diffusivity |
| *d* | 5.5 mm |  |
| *d*_1_ | 5.5 mm |  |
| *d*_2_ | 1.5 mm |  |
| Δ*t* | 0.01 d |  |
| *V*_res_ | 8.33 ml |  |
|  | 75 μl | Initial volume |
| *l*_1_ | 1.5 mm |  |
| *l*_2_ | 6.6 mm |  |

Numerical results of DOC and nitrate concentrations were scaled by dividing with *C*_DOC_ in the center well on day 14 (denoted as *C*_DOC,max_) and *C*_N_ on day 0 (denoted as *C*_N,max_), respectively. Figure 4 and Supplementary Figure S5 were plotted as function of the distance from the center well, normalized by the side length of a microplate. The calculations were performed by using MATLAB (Mathworks).

**References**

1. Samo TJ, Kimbrel JA, Nilson DJ, Pett‐Ridge J, Weber PK, Mayali X. Attachment between heterotrophic bacteria and microalgae influences symbiotic microscale interactions. *Environ Microbiol* 2018; **20**: 4385–4400.

2. Diner RE, Schwenck SM, McCrow JP, Zheng H, Allen AE. Genetic manipulation of competition for nitrate between heterotrophic bacteria and diatoms. *Front Microbiol* 2016; **7**.

3. Refojo MF. Permeation of water through some hydrogels. *J Appl Polym Sci* 1965; **9**: 3417–3426.

4. Seymour JR, Amin SA, Raina J-B, Stocker R. Zooming in on the phycosphere: The ecological interface for phytoplankton–bacteria relationships. *Nat Microbiol* 2017; **2**: 17065.

5. Bjørrisen PK. Phytoplankton exudation of organic matter: Why do healthy cells do it? *Limnol Oceanogr* 1988; **33**: 151–154.

6. Hellebust JA. Excretion of some organic compounds by marine phytoplankton. *Limnol Oceanogr* 1965; **10**: 192–206.

7. Biddanda B, Benner R. Carbon, nitrogen, and carbohydrate fluxes during the production of particulate and dissolved organic matter by marine phytoplankton. *Limnol Oceanogr* 1997; **42**: 506–518.

8. Fukao T, Kimoto K, Kotani Y. Production of transparent exopolymer particles by four diatom species. *Fish Sci* 2010; **76**: 755–760.

9. Passow U. Production of transparent exopolymer particles (TEP) by phyto- and bacterioplankton. *Mar Ecol Prog Ser* 2002; **236**: 1–12.

10. Jackson GA. Simulating chemosensory responses of marine microorganisms. *Limnol Oceanogr* 1987; **32**: 1253–1266.

11. Ge Z, Girguis PR, Buie CR. Nanoporous microscale microbial incubators. *Lab Chip* 2016; **16**: 480–488.

12. Kirchman DL. The uptake of inorganic nutrients by heterotrophic bacteria. *Microb Ecol* 1994; **28**: 255–271.

13. Mullin MM, Sloan PR, Eppley RW. Relationship between Carbon Content, Cell Volume, and Area in Phytoplankton. *Limnol Oceanogr* 1966; **11**: 307–311.
